# Benefit of Panoramic Radiography in the Detection of Carotid Calcifications: Clinical Case Reports and Review of the Literature

**DOI:** 10.1155/2023/3989502

**Published:** 2023-06-03

**Authors:** Olfa Zaghden, Rawia Jaziri, Rym Kammoun, Imen Chaabani, Touhami Ben Alaya

**Affiliations:** ^1^Department of Radiology, University Dental Clinic, University of Monastir, Monastir, Tunisia; ^2^Laboratory of Histology and Embryology, Faculty of Dental Medicine of Monastir, University of Monastir, Monastir, Tunisia; ^3^ABCDF Laboratory for Biological Clinical and Dento-Facial Approach, University of Monastir, Monastir, Tunisia; ^4^Unity of Bioactive Natural Substances and Biotechnology, Faculty of Dental Medicine, University of Monastir, Monastir, Tunisia

## Abstract

Atherosclerotic lesions in the common carotid artery are one of the most frequent causes of ischemic stroke. They are usually diagnosed by cardiologists and are managed following complementary examinations. In dental practice, panoramic radiograph is a basic examination frequently performed in first line. On this radiography, possible unilateral or bilateral opacities projecting to the latero-cervical regions can be visualized, raising suspicion of carotid calcifications. The aim of this study was to present, through three cases and a review of the literature, the benefit of PR in the diagnosis of carotid calcifications and the approaches to be followed in case of such suspicious images. This would contribute, in some cases, to an early diagnosis and management, thus avoiding the evolution towards cerebral vascular accidents.

## 1. Introduction

Atherosclerosis is a multifactorial chronic inflammatory disease. It is characterized by the presence of atheromatous plaques that may be calcified and by the thickening and loss of elasticity of the arterial wall [1]. The first consequence of atheromatous plaque formation is arterial caliber narrowing and carotid stenosis. The latter evolves latently but can eventually lead to stroke, which is the third leading cause of death in most countries [1].

Any method of diagnosis allowing early detection of this pathology is of great benefit for the patient as it leads to its early management, thus preventing its evolution towards possible complications.

Panoramic radiography (PR) is a common imaging technique used for diagnostic and treatment purposes in dental practice [2]. In addition to exploring the dento-maxilla, it allows visualization of the lateral cervical regions, which may be the site of calcifications, particularly carotid ones.

Friedlander and Lande published for the first time the possibility of identifying atheromatous calcifications in the carotid artery on PRs as part of routine dental diagnosis [3, 4]. Early diagnosis leads to an adapted management, thus preventing possible complications that may affect the patients' quality of life or even put at risk the vital prognosis.

The aim of this study was to highlight the benefit of PR in the detection of carotid artery calcifications (CACs) through three cases, discovered incidentally during routine radiological examination, and the approach to be followed by dental practitioners when faced with such possible suspicious images. This would contribute to early diagnosis and management of CACs, thus preventing its evolution towards strokes.

## 2. Cases Presentation

### 2.1. Clinical Case 1

A 74-year-old female patient with diabetes and hypertension, receiving oral antidiabetic and antihypertensive medication, presented to her dentist for temporomandibular joint (TMJ) pain.

**Figure 1:**
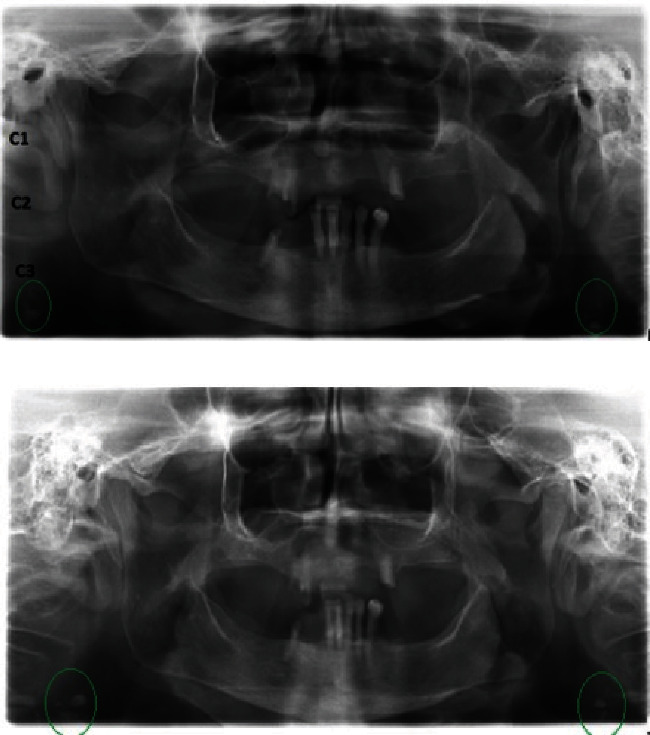
(a) Panoramic radiograph: bilateral irregular radio-opaque images in the carotid areas, behind the mandibular angle, at the same level as the third cervical vertebra (C3; green circle). (b) Panoramic radiograph in anterior translation position, allowing better visibility of the latero-cervical opacities.

First intention PR was performed and it fortuitously revealed bilateral calcifications in the third cervical vertebra (C3), behind the mandibular angle and adjacent to the hyoid bone (Figure 1(a)).

To allows better visualization of the cervical spine and lateral regions, and consequently better visibility of the suspected calcifications, a second PR in the anterior translation position of the arches was performed (Figure 1(b)).

**Figure 2:**
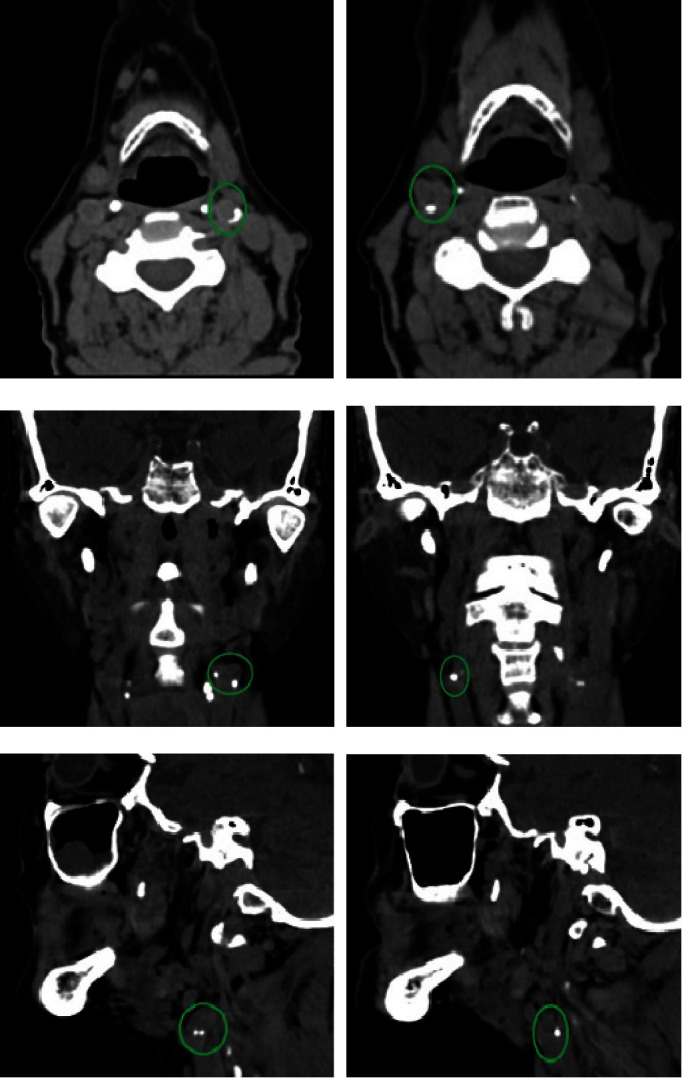
(a) and (b) Axial narrow window CT sections without injection of contrast medium through the third cervical vertebra, revealing hyperdensities peripheral to the carotid artery, located posterolateral to the pharyngeal area, having different shapes: curvilinear “C” shape (a) and globular homogeneous (b). (c) and (d) Coronal CT sections in narrow window without injection of contrast medium through the third cervical vertebra, revealing hyperdensities located laterally to the third cervical vertebra with globular appearance. (e) and (f) Sagittal reconstructions through the cervical hyperdensities, revealing globular calcifications in front of the anterior tubercles of the cervical vertebrae.

To explore TMJ and to clarify the location and nature of these calcifications, computed tomography (CT) scan was performed. This examination revealed bilateral hyperdense images located in the periphery of the carotid arteries, extending vertically for approximately 5 mm, and presenting different shapes depending on the plane and level of the section (Figures 2(a), 2(b), 2(c), 2(d), 2(e), and 2(f)).

#### 2.1.1. Axial Sections

Figures 2(a) and 2(b) revealed bilateral homogeneous, globular calcifications in the right carotid artery and “C”-shaped curvilinear calcifications in the left carotid artery. These calcifications were located in the soft tissues posterolateral to the pharyngeal area.

#### 2.1.2. Coronal Sections

Figures 2(c) and 2(d) revealed bilateral calcifications with linear and/or globular appearance, located laterally to the third cervical vertebra (C3).

#### 2.1.3. Sagittal Sections

Figures 2(e) and 2(f) revealed coalescing globular calcifications, located in the soft tissue anterior to the anterior tubercles of the transverse processes of the third cervical vertebra (C3).

Based on imaging data (PR and CT), diagnosis of bilateral calcification of the common carotid artery was strongly suspected.

The patient was lost to follow-up, and further evaluation of the severity of carotid calcifications was not possible.

### 2.2. Clinical Case 2

A 70-year-old female patient, hypertensive and well-followed by her cardiologist, consulted her dentist for implant-supported prosthetic rehabilitation.

A pre-implant radiological assessment, including PR and cone-beam computed tomography (CBCT) examination was performed.

**Figure 3:**
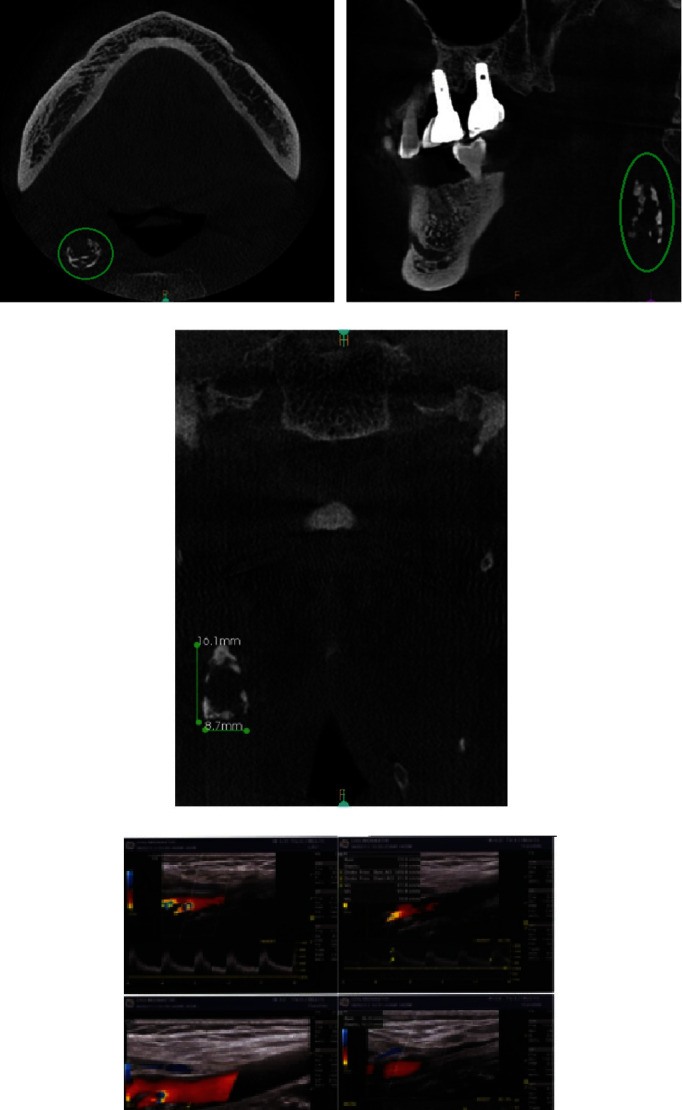
(a) Panoramic radiograph revealing unilateral irregular radiopaque masses behind the right mandibular angle between C2 and C3 (green circle). (b) Cone beam axial section revealing homogeneous linear and curvilinear calcifications, located posterolateral to the pharyngeal area. (c) Cone beam sagittal section revealing calcifications with a coalescent globular appearance, anterior to the anterior tubercles of the cervical vertebrae. (d) Cone beam coronal section revealing calcifications with a linear, linear-globular appearance, located laterally in the soft tissue. (e) Cervico-encephalic Doppler ultrasound.

During the analysis of the radiological assessment, and by carefully reading PR at the level of the right latero-cervical region, multiple coalescing, heterogeneous, radiopaque images of calcific tone with irregular boundaries, projecting at the level of the prevertebral soft tissues, between the second and third cervical vertebrae (C2 and C3), posterior to the mandibular angle, adjacent to the hyoid bone, and extending vertically for approximately 16 mm were detected (Figure 3(a)).

CBCT allowed for more precise verification of the location and nature of the calcifications visualized on PR.

Figure 3(b) revealed multiple homogeneous, curvilinear calcifications, coalescing into a large “C.” These calcifications were located in the soft tissue posterolateral to the pharyngeal area.

Figure 3(c) revealed vertical and oblique calcifications having the appearance of coalescing globules. These calcifications were located in the soft tissue facing the anterior tubercles of the cervical vertebrae.

Figure 3(d) revealed multiple calcifications with linear and/or linear-globular aspect. These calcifications were located laterally in the soft tissue.

Based on these imaging data (PR and CBCT), diagnosis of a possible calcification of the right carotid artery was made. The patient was referred to her cardiologist who completed the already performed radiological assessment with a cervico-encephalic Doppler ultrasound (CEDU; Figure 3(e)).

CEDU revealed an atheromatous-calcific infiltration of the internal carotid arteries mainly at the level of the bulbs, particularly an extended right carotid bulb plaque at the origin of the internal carotid artery of 14 mm in long axis and of maximum thickness of 6 mm, with a heterogeneous echo-structure, a seat of calcifications with irregular internal contours responsible for a reduction in arterial surface area, having an acceleration of circulatory velocities reaching 150 cm/s downstream.

Cardiac ultrasound was also included in physical examination. It revealed non-obstructive left ventricular hypertrophy, with maintenance of excellent left ventricular systolic function.

After completion of the cardiological examination, stenosis was assessed as non-obstructive. Medical treatment and regular check-ups were indicated for this patient.

## 3. Clinical Case 3

A 79-year-old patient, declaring to have no particular history, complained of transient loss of consciousness during lateral head rotation, radiating neck pain, and limited lateral movements during prosthetic rehabilitation sessions.

**Figure 4:**
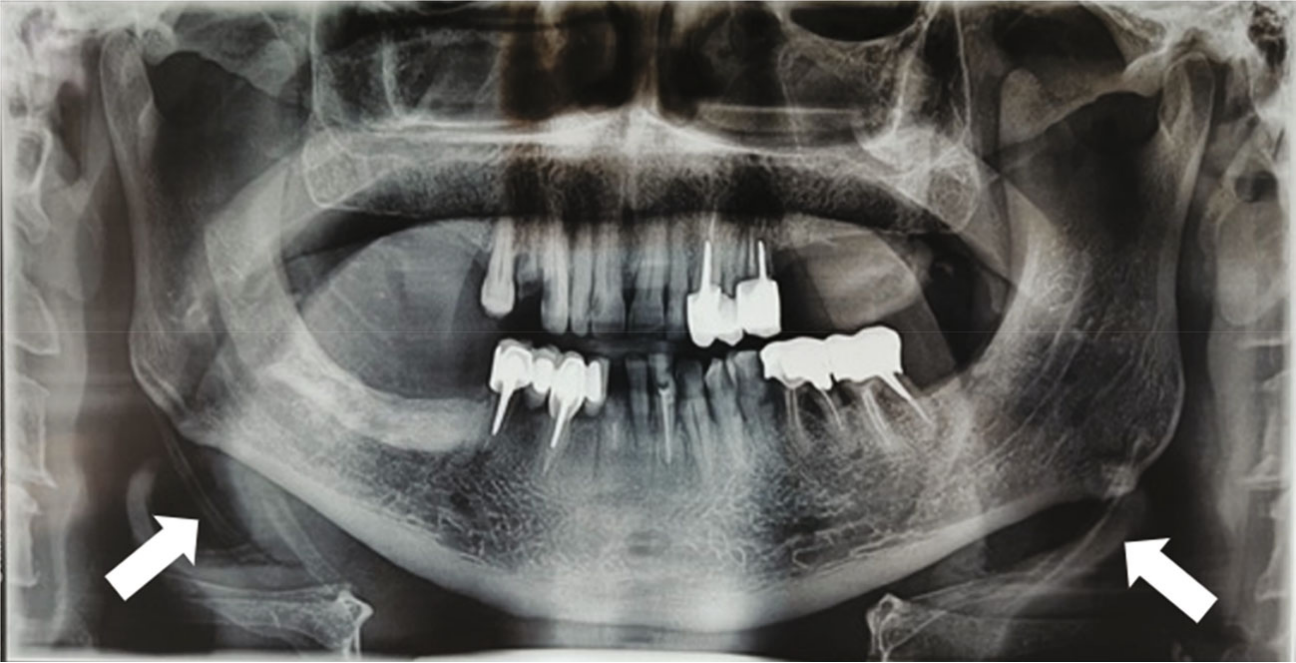
Panoramic radiograph revealing bilateral calcification of the stylohyoid ligaments (arrows).

First-line PR was performed and it fortuitously revealed the presence of bilateral stylohyoid ligament calcifications (Figure 4).

The preliminary diagnosis was Eagle's syndrome.

**Figure 5:**
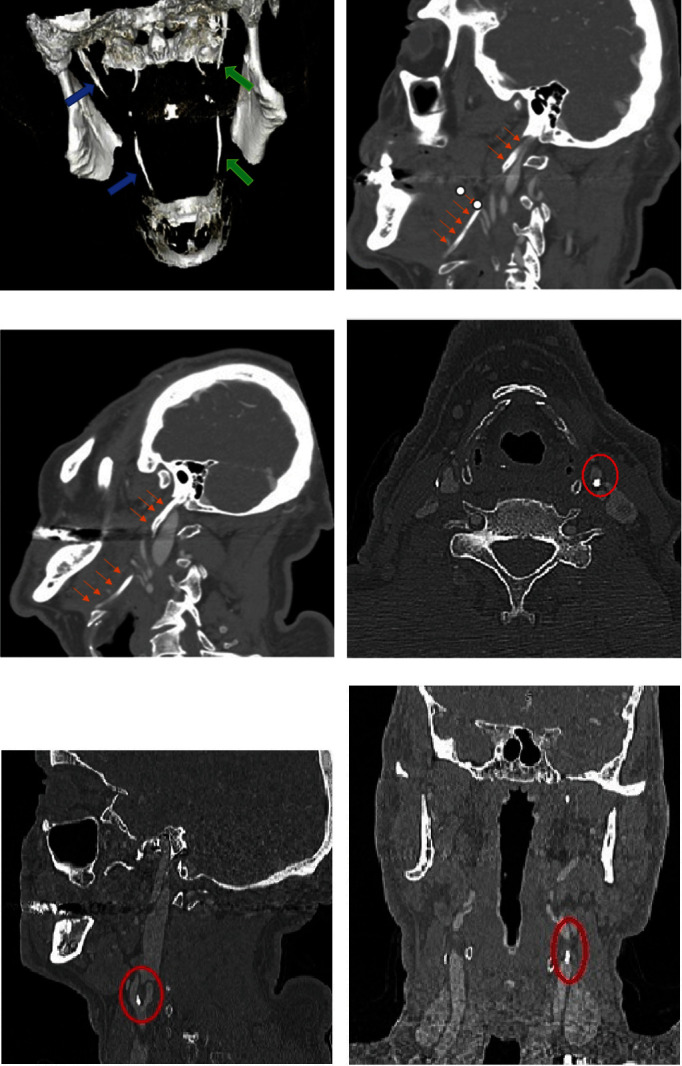
(a) Cervicofacial CT: 3D reconstruction in posterior view revealing bilateral calcification of the stylohyoid ligaments (right side: blue arrows and left side: green arrows). (b) and (c) Cervicofacial CT: sagittal reconstruction—ossified aspect of the stylohyoid ligaments [right (b) and left (c)] giving the appearance of a long styloid process (arrows). (d) Axial section revealing calcification of roughly rounded appearance with irregular contour, located posterolaterally to the pharyngeal area. (e) Sagittal reconstruction revealing calcification at the level of the left carotid bulb, having an appearance of coalescing globules in front of the anterior tubercles of the fifth cervical vertebra (C5). (f) Coronal reconstruction revealing calcification of the common carotid artery with a linear-globular appearance.

Cervicofacial CT scan was performed and it showed left carotid calcifications in addition to bilateral calcifications of the stylohyoid ligaments (Figures 5(a), 5(b), 5(c), 5(d), 5(e), and 5(f)).

**Figure 6:**
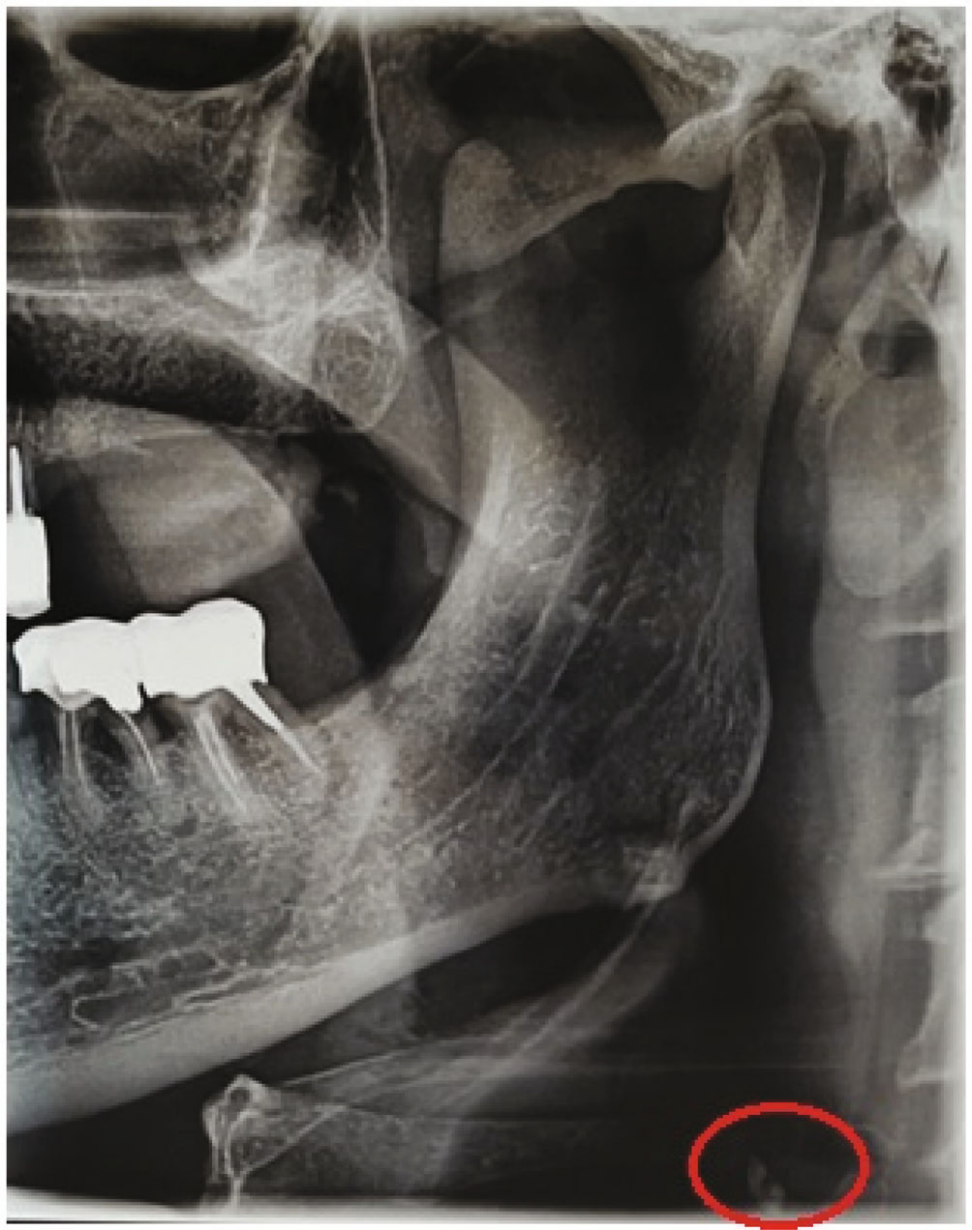
A cropped and magnified image of Figure 4. Panoramic radiograph on the left side, revealed radiopacity of calcific tone (red circle), projecting to the pre-vertebral soft tissues, opposite to the fifth cervical vertebra (C5).

A careful rereading of PR (Figure 6) revealed radioopacity of calcific tone, with irregular boundaries, projecting to the pre-vertebral soft tissues, opposite to the fifth cervical vertebra (C5), behind the mandibular angle and adjacent to the hyoid bone, located at the level of the left latero-cervical region.

The patient's extensive history revealed that he had hypercholesterolemia, and he had been diagnosed with acute coronary syndrome ST (−) with an electrically positive stress test in 2012, leaving heart failure as a sequela.

The patient reported that he had stopped taking his medication 8 years earlier.

**Figure 7:**
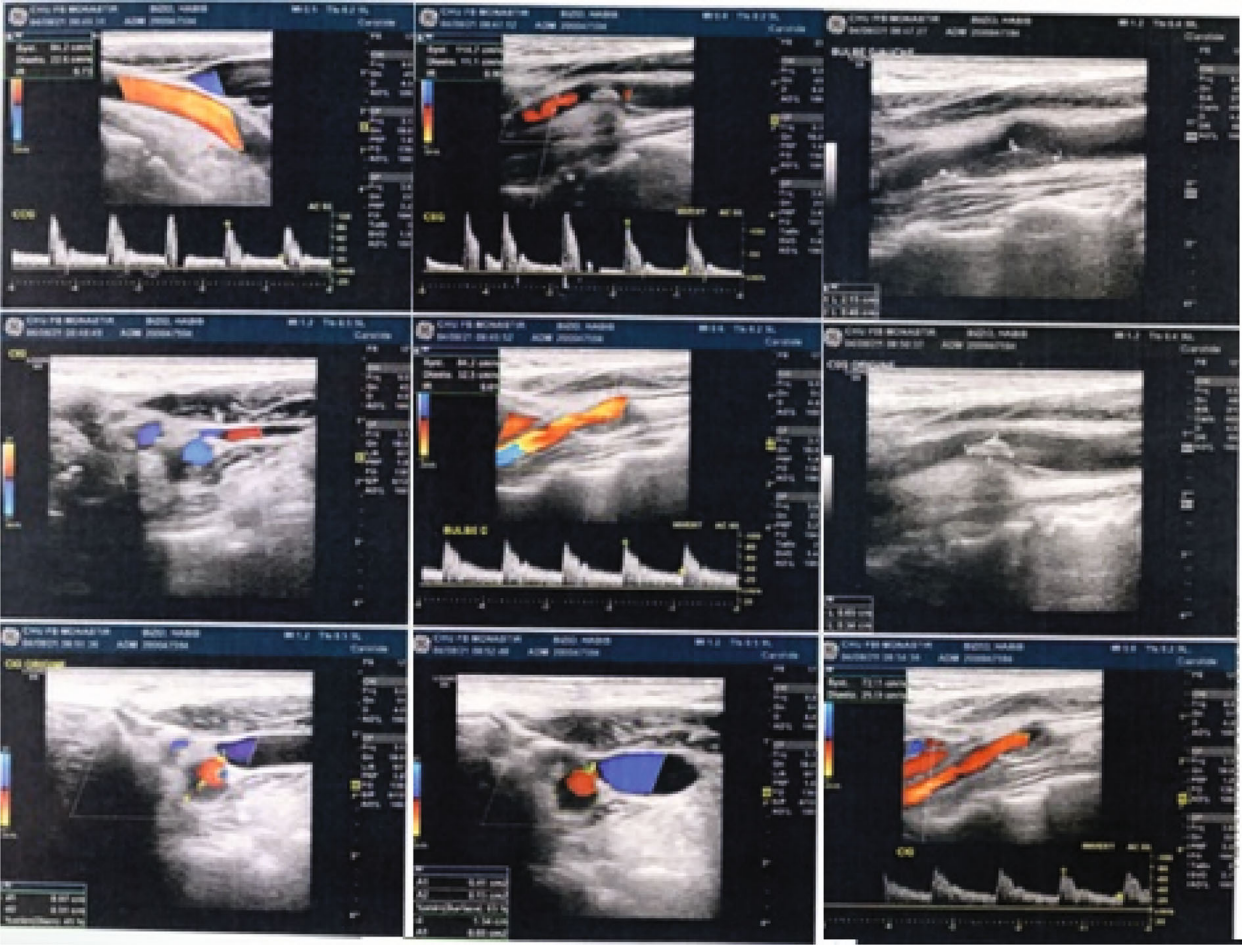
Cervico-encephalic Doppler ultrasound.

Based on these clinical data, he was referred to his cardiologist, and the radiological assessment was completed with CEDU (Figure 7). The latter confirmed the presence of atheromatous-calcific plaques at the level of the extended left carotid bulb at the origin of the internal carotid artery, responsible for a stenosis of 40% in diameter.

## 4. Discussion

Stroke is a major public health problem, representing the third leading cause of death in industrialized countries after heart disease and cancer. It is also the most frequent cause of acquired disability in adulthood [1, 5].

Each year, in Tunisia, approximately 192 out of 100,000 inhabitants have a cerebrovascular accident. Among these strokes, 79.5% are of ischemic origin, and 20.5% are of hemorrhagic origin [6].

Ischemic lesions may have several etiologies, with carotid atherosclerosis being one of them.

Friedlander and Friedlander defined the apex of the common carotid bifurcation and the posterior wall of the proximal portion of the internal carotid artery as the regions most frequently affected by atheromatous lesions [7].They used the term carotid artery territory (CAT) to determine the region of projection of the common carotid artery on the images. It is limited anteriorly by the ascending branch of the mandible and the mandibular angle, and posteriorly by the bodies of the second, third, and fourth cervical vertebrae (C2–C3–C4) [7]. They also described the lower border of the body of the third cervical vertebra (C3), which is located 1.5–4 cm below and behind the mandibular angle, as another typical projection area. On PR, calcifications are indeed located along this vertical vascular pathway [7].

Radiologically, calcified atheromatous plaques are presented as circular, irregular or heterogeneous, and unilateral or bilateral radio-opacities. These plaques have different shapes. They may have a predominantly circular appearance when they are small; however, large atheromas have a linear or thin rectangular appearance [8].

The usual location of these calcifications is inferiorly and posteriorly to the angle of the mandible [9], opposite, above, or just below the intervertebral space between the third and the fourth cervical vertebrae (C3 and C4) [10], and close to the hyoid bone [9].

In the first clinical case, these calcifications had the aspect of globular radio-opaque images, bilateral, overlying the prevertebral soft tissues, and located at the same level as the third cervical vertebra (C3) behind the mandibular angle and adjacent to the hyoid bone.

In the second case, these calcifications had the appearance of multiple irregular, linear, rectangular, and unilateral radiopaque images. These radio-opacities were overlying the prevertebral soft tissues and located between the second and the third cervical vertebrae (C2 and C3) behind the mandibular angle and adjacent to the hyoid bone.

In the third case, the calcifications had the aspect of a roughly rounded and ill-defined radiopacity, overlying the prevertebral soft tissue, and located at the same level as the fifth cervical vertebra (C5).

To establish diagnosis, it is important to differentiate carotid calcifications from other radio-opacities. These radiopacities may be anatomic or pathologic.

The adjacent anatomical structures that may be overlapping in the carotid area on PR include the hyoid bone, calcification of the triticeal cartilage, the superior horn of the thyroid cartilage, the stylohyoid ligament, and the epiglottis [11–14].

The main pathological calcifications of adjacent soft structures that can also be confused with CAC are the calcifications of the palatine tonsils, phleboliths, lithiasis of the main salivary glands, rhinoliths, and the calcifications of the cervical lymph nodes [11–14].

Therefore, correct identification of CACs on PR is not an easy task, especially in the context of an asymptomatic patient undergoing oral exploration.

The reliability of PR in detecting CAC is still a controversial topic. It is confirmed by the study of diagnostic agreement between PR and CEDU. The latter is considered the “gold standard” in the diagnosis of stenosis [15, 16], but its use to screen a large asymptomatic population for early diagnosis is not cost-effective due to its high cost [11, 16, 17].

Several authors have investigated agreement between PR and CEDU, and they determined the level of agreement by calculating Cohen's kappa coefficient (*κ*). [11, 18–24]. Khosropanah et al. classified it as *κ* < 0.4 is poor; *κ* = 0.4–0.75 is fair to good; and *κ* > 0.75 is very good to excellent [18].

The agreement values reported in the literature are varied. Some authors consider PR to have good (70%) to excellent (80%) accuracy [11, 19–24] and to have high level of agreement with CEDU [11, 19]. The largest agreement value in the literature is described by Khambete et al. The *κ* value calculated in this study was found to be equal to 0.8. Statistically, this value is interpreted as “almost perfect agreement” [25]. Other authors have reported moderate level of agreement with *κ* value of 0.61 [26]. However, others have suggested that PR has poor agreement with CEDU and reported *κ* values in the range of 0.488 [10] and 0.27 [18], and thus reported that PR has a significant limitation in the detection of CAC [5, 9].

The reliability of PR is characterized by its sensitivity, specificity, positive predictive value (PPV), and negative predictive value (NPV; see Appendix).

In the literature, the sensitivity of PR varies between 31.1% and 100% [5, 11, 19, 26–30].

Low sensitivity and the large number of false negatives may be the repercussion of several elements, such as the superposition of carotid calcifications on the surrounding anatomical elements [5], a small size with a low degree of calcification [30], the variability in the location of the bifurcation of the common carotid artery that can project outside the field of PR exploration, and the errors in patients' positioning during X-ray exposure [30].

Some authors report a significant amount of relative subjectivity in reading PRs, thus training PR examiners significantly improves the sensitivity of the test [21].

Similarly, the specificity of PR is variable from one study to another [9, 30].

The large variation in results reflects the difference in the study protocols chosen by different authors. Indeed, when the baseline radiological examination (CEDU) is performed before PR or examined retrospectively [5, 18, 31], the sensitivity of PR is low (22.2–66.6%), whereas its specificity remains high (48–90%). This is probably because the studies were not limited to the lesions located in the exploration field of PR. CEDU has a wider field of exploration than that of PR, thus increasing the number of false negatives.

However, if the vascular examination is performed following a fortuitous discovery of a radiopacity in the carotid area visible on PR, as described in the studies of Bastos and Abreu and Ertas and Sisman, the sensitivity of PR for this indication is significantly improved [17, 19].

Yet, the clinical significance of incidental calcification in the carotid area on PR is uncertain, especially in the context of an asymptomatic patient. Therefore, it is important to ask the following question: Does this incidental calcification visible on PR require further evaluation?

Constantine et al. reported that carotid calcification visible on PR has 15% probability of reflecting clinically significant stenoses (≥50%) on CEDU [32].

Almog et al. demonstrated that 50% of patients having CAC on their PRs have carotid artery stenosis greater than 50%, and when PRs do not show calcification, the percentage of stenosis is about 21% [33].

According to Johansson et al., 7–23% of incidentally discovered CACs on PRs reflect significant stenosis of the carotid artery, which is strongly associated with a risk of stroke and myocardial infarction [34].

A more thorough clinical evaluation is therefore required, and dentists should carefully examine CAT on routine PRs, especially in elderly patients with risk factors, such as hypertension, diabetes mellitus, obesity, hyperlipidemia, and smoking [12, 20, 35]. Early detection could prevent cerebral vascular accidents and improve patients' vital prognosis. Indeed, Cohen et al. reported that 32.4% of patients with CAC diagnosed by PR were victims of cerebral vascular accidents (myocardial infarction, stroke, transient ischemic attack, and angina) [12].

These results are consistent with those reported by Bengtsson et al., demonstrating that patients, aged 60–96 years, with calcified carotid plaque detectable by PR are more susceptible to stroke and/or ischemic heart disease [36].

The data presented in this work confirm that PR is not as useful as CEDU and CT for confirming or follow-up CACs. It cannot therefore be considered as a routine screening tool for patients with carotid atheromatous lesions, whatever is the degree of stenosis, due to the interpretation variability between different dentists, the anatomic variability, the patients' positioning, and the apparatus settings. However, this non-invasive method, which is more accessible compared with other imaging techniques, has great potential for early detection of CAC in patients with or without associated risk factors.

MacDonald et al. proposed a therapeutic approach to be followed by dentists when faced with such possible suspicious images [37]. This approach is guided by the patient's general condition and medical history. Thus, detection of CAC by the dentist leads to two different therapeutic approaches. Detection of a radiopacity compatible with CAC in an asymptomatic patient with no medical or surgical history, especially if the patient is young should prompt the dental practitioner to demand additional examinations in order to search for a general disease, such as diabetes mellitus, and/or to refer the patient to the appropriate physician. For patients with known and monitored cardiovascular risk factors, the approach is different. Dental practitioners should contact the patient's treating physician to determine whether or not the patient should be referred.

Thus, incidental detection of carotid calcifications on PR can potentially increase the length and quality of life, especially in patients with symptomatic carotid stenosis, through early management. Therefore, dentists need to pay more attention to the latero-cervical areas, much overlooked on PR.

To conclude, management of carotid stenosis is multidisciplinary, involving the dentist, the patient's treating physician, the radiologist, the cardiologist, and the vascular surgeon. The dentist is one of the key elements in this chain. Sometimes, he intervenes in the first line by initially detecting carotid calcifications on PR and thus contributing to an early diagnosis and management avoiding the evolution towards strokes. Thus, this radiological examination has great potential for early detection of CAC in patients with or without associated risk factors and serves as a support for the usual techniques of carotid calcification screening.

## Data Availability

Data supporting this case report are available from the corresponding author or first author on reasonable request.

## Appendix

### Evaluation Criteria for Diagnostic Tests

#### Positive Predictive Value (PPV)

It determines the ability of the panoramic X-ray to confirm, when positive, the presence of CAC. It indicates the probability of the disease occurrence when the test result is positive.

#### Negative Predictive Value (NPV)

It determines the capacity of panoramic X-ray to confirm, when negative, the presence of CAC. It indicates the probability that the disease is absent when the test result is negative.

#### Sensitivity

It is the probability of detecting radiopacity in the carotid area, compatible with calcification of the carotid artery on panoramic X-ray when it exists on the reference examination.

#### Specificity

It is the probability of not detecting radiopacity in the carotid area when it does not exist on the reference examination.

## References

[1] Moshfeghi M., Beigom Taheri J., Bahemmat N., Ebrahim Evazzadeh M., Hadian H. (2014). Relationship between carotid artery calcification and dental panoramic images, hypertension and myocardial infarction. *Iranian Journal of Radiology*.

[2] The use of dental radiographs (2006). The use of dental radiographs. *Journal of the American Dental Association*.

[3] Friedlander A. H., Lande A. (1981). Panoramic radiographic identification of carotid arterial plaques. *Oral Surgery, Oral Medicine, and Oral Pathology*.

[4] Santos J., Soares G., Alves A., Kurita L., Silva P., Costa F. (2018). Prevalence of carotid artery calcifications among 2,500 digital panoramic radiographs of an adult Brazilian population. *Medicina Oral, Patologia Oral y Cirugia Bucal*.

[5] Madden R. P., Hodges J. S., Salmen C. W. (2007). Utility of panoramic radiographs in detecting cervical calcified carotid atheroma. *Oral Surgery, Oral Medicine, Oral Pathology and Oral Radiology*.

[6] Moalla K. S., Damak M., Chakroun O. (2020). Facteurs pronostiques de mortalité par accident vasculaire cérébral artériel à la phase aiguë dans une population nord-africaine. *The Pan African Medical Journal*.

[7] Friedlander A. H., Friedlander I. K. (1998). Identification of stroke prone patients by panoramic radiography. *Australian Dental Journal*.

[8] Ahmad M., Madden R., Perez L. (2005). Triticeous cartilage: prevalence on panoramic radiographs and diagnostic criteria. *Oral Medicine, Oral Pathology and Oral Radiology Endodontology*.

[9] Alves N., Deana N. F., Garay I. (2014). Detection of common carotid artery calcifications on panoramic radiographs: prevalence and reliability. *International Journal of Clinical and Experimental Medicine*.

[10] Akkemik O., Kazaz H., Tamsel S., Dündar N., Sahinalp S., Ellidokuz H. (2020). A 5 years follow-up for ischemic cardiac outcomes in patients with carotid artery calcification on panoramic radiographs confirmed by Doppler ultrasonography in Turkish population. *Dentomaxillofacial Radiology*.

[11] Romano-Sousa C. M., Krejci L., Medeiros F. M. M. (2009). Diagnostic agreement between panoramic radiographs and color Doppler images of carotid atheroma. *Journal of Applied Oral Science*.

[12] Cohen S. N., Friedlander A. H., Jolly D. A., Date L. (2002). Carotid calcification on panoramic radiographs: an important marker for vascular risk. *Oral Medicine, Oral Pathology and Oral Radiology Endodontology*.

[13] Friedlander A. H., Garrett N. R., Norman D. C. (2002). The prevalence of calcified carotid artery atheromas on the panoramic radiographs of patients with type 2 diabetes mellitus. *Journal of the American Dental Association (1939)*.

[14] Abreu T., Ferreira E., de Brito F. S., de Sales K. F., Lopes F., de Oliveira A. F. (2015). Prevalence of carotid artery calcifications detected on panoramic radiographs and confirmed by Doppler ultrasonography: their relationship with systemic conditions. *Indian Journal of Dental Research*.

[15] Almog D. M., Illig K. A., Khin M., Green R. M. (2000). Unrecognized carotid artery stenosis discovered by calcifications on a panoramic radiograph. *Journal of the American Dental Association*.

[16] Schroder A. G. D., de Araujo C. M., Guariza-Filho O., Flores-Mir C., de Luca C. G., Porporatti A. L. (2019). Diagnostic accuracy of panoramic radiography in the detection of calcified carotid artery atheroma: a meta-analysis. *Clinical Oral Investigations*.

[17] Bastos J. S., Abreu T. Q. (2012). Sensitivity and accuracy of panoramic radiography in identifying calcified carotid atheroma plaques. *Brazilian Journal of Oral Sciences*.

[18] Khosropanah S. H., Shahidi S. H., Bronoosh P., Rasekhi A. (2009). Evaluation of carotid calcification detected using panoramic radiography and carotid Doppler sonography in patients with and without coronary artery disease. *British Dental Journal*.

[19] Ertas E. T., Sisman Y. (2011). Detection of incidental carotid artery calcifications during dental examinations: panoramic radiography as an important aid in dentistry. *Oral Surgery Oral Medicine Oral Pathology Oral Radiology Endodontology*.

[20] Atalay Y., Asutay F., Agacayak K. S., Koparal M., Adali F., Gulsun B. (2015). Evaluation of calcified carotid atheroma on panoramic radiographs and Doppler ultrasonography in an older population. *Clinical Interventions in Aging*.

[21] Yoon S. J., Yoon W., Kim O. S., Lee J. S., Kang B. C. (2008). Diagnostic accuracy of panoramic radiography in the detection of calcified carotid artery. *Dentomaxillofacial Radiology*.

[22] Abecasis P., Chimenos-Kustner E., Lopez-Lopez J. (2014). Orthopantomography contribution to prevent ischemic stroke. *Journal of Clinical and Experimental Dentistry*.

[23] Alman A. C., Johnson L. R., Calverley D. C., Grunwald G. K., Lezotte D. C., Hokanson J. E. (2013). Validation of a method for quantifying carotid artery calcification from panoramic radiographs. *Oral Surgery, Oral Medicine, Oral Pathology, Oral Radiology*.

[24] Yeluri G., Ca K., Raghav N. (2015). Correlation of dental pulp stones, carotid artery and renal calcifications using digital panoramic radiography and ultrasonography. *Contemporary Clinical Dentistry*.

[25] Khambete N., Kumar R., Risbud M., Joshi A. (2014). Reliability of digital panoramic radiographs in detecting calcified carotid artery atheromatous plaques: a clinical study. *Indian Journal of Dental Research*.

[26] Ravon N. A., Hollender L. G., Mcdonald V., Persson G. R. (2003). Signs of carotid calcification from dental panoramic radiographs are in agreement with Doppler sonography results. *Journal of Clinical Periodontology*.

[27] Friedlander A. H., Garrett N. R., Chin E. E., Baker J. D. (2005). Ultrasonographic confirmation of carotid artery atheromas diagnosed via panoramic radiography. *Journal of the American Dental Association*.

[28] Bayram B., Uckan S., Acikgoz A., Müderrisoğlu H., Aydinalp A. (2006). Digital panoramic radiography: a reliable method to diagnose carotid artery atheromas?. *Dentomaxillofacial Radiology*.

[29] Imanimoghaddam M., Rah Rooh M., Mahmoudi Hashemi E., Javadzade B. A. (2012). Doppler sonography confirmation in patients showing calcified carotid artery atheroma in panoramic radiography and evaluation of related risk factors. *Journal of Dental Research, Dental Clinics, Dental Prospects*.

[30] Bengtsson V. W., Persson G. R., Renvert S. (2014). Assessment of carotid calcifications on panoramic radiographs in relation to other used methods and relationship to periodontitis and stroke: a literature review. *Acta Odontologica Scandinavica*.

[31] Damaskos S., Griniatsos J., Tsekouras N. (2008). Reliability of panoramic radiograph for carotid atheroma detection: a study in patients who fulfill the criteria for carotid endarterectomy. *Oral Medicine, Oral Pathology and Oral Radiology Endodontology*.

[32] Constantine S., Roach D., Liberali S. (2019). Carotid artery calcification on orthopantomograms (CACO study) – is it indicative of carotid stenosis?. *Australian Dental Journal*.

[33] Almog D. M., Horev T., Illig K. A., Green R. M., Carter L. C. (2002). Correlating carotid artery stenosis detected by panoramic radiography with clinically relevant carotid artery stenosis determined by duplex ultrasound. *Oral Medicine, Oral Pathology and Oral Radiology Endodontology.*.

[34] Johansson E., Ahlqvist J., Garoff M., Levring Jäghagen E., Meimermondt A., Wester P. (2015). Carotid calcifications on panoramic radiographs: a 5-year follow-up study. *Oral Surgery, Oral Medicine, Oral Pathology, Oral Radiology*.

[35] Levy C., Mandel L. (2010). Calcified carotid artery imaged by computed tomography. *Journal of Oral and Maxillofacial Surgery*.

[36] Bengtsson V. W., Persson G. R., Berglund J., Renvert S. (2019). Carotid calcifications in panoramic radiographs are associated with future stroke or ischemic heart diseases: a long-term follow-up study. *Clinical Oral Investigations*.

[37] MacDonald D., Chan A., Harris A., Vertinsky T., Farman A. G., Scarfe W. C. (2012). Diagnosis and management of calcified carotid artery atheroma: dental perspectives. *Oral Surgery, Oral Medicine, Oral Pathology, Oral Radiology*.

